# South American special issue: Editorial

**DOI:** 10.1002/prp2.868

**Published:** 2021-10-05

**Authors:** Federico Monczor, Ana Genaro

**Affiliations:** ^1^ Facultad de Farmacia y Bioquímica Instituto de Investigaciones Farmacológicas (ININFA‐UBA‐CONICET) Universidad de Buenos Aires Buenos Aires Argentina; ^2^ Instituto de Investigaciones Biomédicas (UCA‐CONICET) Buenos Aires Argentina; ^3^ Departamento de Farmacología Facultad de Medicina UBA Paraguay Buenos Aires Argentina

Pharmacology is essentially an interdisciplinary science. As such, knowledge gained in several different areas is gathered to focus on human health. Although South America is a region ravaged by multiple political and economic conflicts throughout its history, cell biology and pharmacological studies were conducted by many South American outstanding researchers working in their own countries and abroad. On the region, Argentine has a privileged history, including Bernardo Houssay, who was awarded the Nobel Prize in Physiology and Medicine in 1947 for his work on pituitary hormones^1^; Luis F. Leloir, who was awarded the Nobel Prize in Chemistry in 1970 for his research on glycogen metabolism^2^; Eduardo Braun‐Menendez, who was one of the discoverers of angiotensin^3^; Adolfo J. De Bold, who discovered atrial natriuretic factor,^4^ and a team that made a great contribution that was pivotal for understanding the presynaptic regulation of neurotransmitter release.^5^, ^6^ All these exceptional researchers and their contributions helped to establish an Argentinean pharmacological tradition that still has a strong influence nowadays. Almost 30% of all researchers recruited by the most important scientific national organism (CONICET) are involved only in biomedical research.

Unfortunately, projects and scientific careers in South America are often finished or significantly delayed because of adverse social conditions, political turmoil, and economic collapses. Unhappily, this has been recurrent in most of our countries and a constant in the region. Nevertheless, there is a pharmacological tradition that continues producing exceptional work contributing to our current knowledge. When economic constraints and infrastructure issues are constant, you are forced to harness your imagination and creativity and make the most of them.

This special issue features articles written by a selected group of leading South American scientists working on different aspects of pharmacology who share the passion for knowledge and use their inspiration to search for alternatives making the most of their budgets and compensating the shortages with innovation.

The common thread of the articles comprised by this issue is the exploration of options or alternative uses for well‐established compounds or systems. With resource optimization as their motivation, Gorzalczany et al.^7^ wrote an article discussing potential substitutes for the use of experimental animals, Giunti et al.^8^ consider the use of a simple and economic platform based on nematodes for drug discovery, and Nuske et al.^9^ explore the application of algorithms and computer simulations to optimize and reduce bioequivalence studies.

On the other hand, examining alternatives for the treatment of well‐studied pathological situations, Nader et al.^10^ study the use of probiotics in hygienic products for the treatment of female urogenital tract infections, Schofs et al.^11^ discuss the antimicrobial effects of Cannabis sativa against fungus and pathogenic bacteria to assess its potential use as an antimicrobial agent, and Formica et al.^12^ report the benefits of novel nanoparticles for the delivery of anti‐vascular endothelial growth factor agents for ocular angiogenesis.

Finally, exploring the complexity of some conditions that require innovative approaches, Palumbo et al.^13^ explore how immunomodulation by neuropeptides can be a valid strategy for the treatment of neurodegenerative disorders, and Sarasola et al.^14^ investigate the immunomodulatory roles of histamine for cancer treatment.

In summary, this special issue of Pharmacology Research & Perspectives will provide an outlook of the current research in South America, hoping that readers will find the articles to be of interest and that help promote collaboration with colleagues in other parts of the world.

## References

[1] The Nobel Prize in Physiology or Medicine 1947. NobelPrize.org. Accessed September 1, 2021. https://www.nobelprize.org/prizes/medicine/1947/summary/

[2] The Nobel Prize in Chemistry 1970. NobelPrize.org. Accessed September 1, 2021. https://www.nobelprize.org/prizes/chemistry/1970/summary/

[3] Braun‐Menendez E . Pharmacology of renin and hypertension. Pharmacol Rev. 1956;8(1):25‐55. 13335488

[4] de Bold AJ . Atrial natriuretic factor: a hormone produced by the heart. Science. 1985;230(4727):767‐770. doi:10.1126/science.2932797. 2932797

[5] Enero MA , Langer SZ , Rothlin RP , Stefano FJ . Role of the alpha‐adrenoceptor in regulating noradrenaline overflow by nerve stimulation. Br J Pharmacol. 1997;120(4 suppl):361‐377; discussion 358‐360. doi:10.1111/j.1476-5381.1997.tb06817.x. 9142415 PMC3224312

[6] Langer SZ . 25 years since the discovery of presynaptic receptors: present knowledge and future perspectives. Trends Pharmacol Sci. 1997;18(3):95‐99. doi:10.1016/s0165-6147(96)01034-6. 9133779

[7] Gorzalczany S , Rodriguez Basso AG . Strategies to apply 3Rs in preclinical testing. Pharmacol Res Perspect. 2021;9:e00863. doi:10.1002/prp2.863. 34609088 PMC8491455

[8] Giunti S , Andersen N , Rayes D , et al. Drug discovery: insights from the invertebrate *C. elegans* . Pharmacol Res Perspect. 2021;9:e00721. doi:10.1002/prp2.721. 33641258 PMC7916527

[9] Nuzke E , Morozov M , Serra H . Genetic algorithms and running simulations from ordinary differential equations based model ‐GA‐RxODE‐ method to optimize bioequivalence studies. Pharmacol Res Perspect. 2021;9:e00824. doi:10.1002/prp2.824. 34609078 PMC8491459

[10] Nader Macías ME , De Gregorio P , Silva J . Probiotic lactobacilli in formulas and hygiene products for the health of the urogenital tract. Pharmacol Res Perspect. 2021;9:e00787. doi:10.1002/prp2.787. 34609059 PMC8491456

[11] Schofs L , Sparo M , Sánchez BS . The antimicrobial effect behind Cannabis sativa. Pharmacol Res Perspect. 2021;9:e00761. doi:10.1002/prp2.761. 33822478 PMC8023331

[12] Formica M , Awde Alfonso H , Palma S . Biological drug therapy for ocular angiogenesis: anti‐VEGF agents and novel strategies based on nanotechnology. Pharmacol Res Perspect. 2021;9:e00723. doi:10.1002/prp2.723. 33694304 PMC7947217

[13] Palumbo M , Moroni A , Quiroga S , et al. Immunomodulation induced by CNS‐related peptides as a therapeutic strategy for neurodegenerative disorders. Pharmacol Res Perspect. 2021;9:e00795. doi:10.1002/prp2.795. 34609083 PMC8491457

[14] Sarasola M , Táquez Delgado M , Nicoud M , et al. Histamine in cancer immunology and immunotherapy. Current status and new perspectives. Pharmacol Res Perspect. 2021;9:e00778. doi:10.1002/prp2.778. 34609067 PMC8491460

